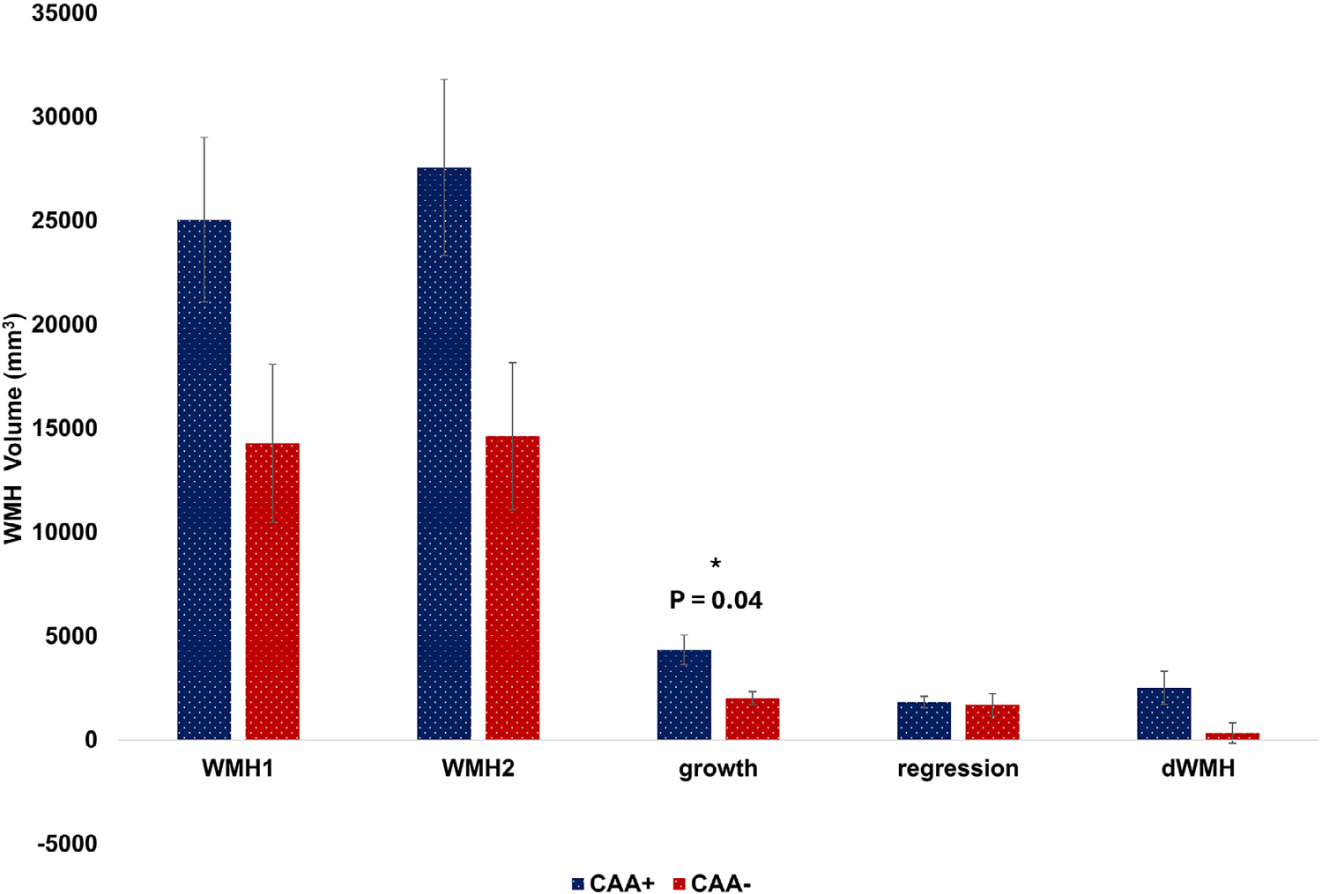# WMH Growth/Regression Computation Method Can Serve as A Sensitive Neuroimaging Biomarker for CAA

**DOI:** 10.1002/alz70856_107274

**Published:** 2026-01-09

**Authors:** Michael Tyler Jessup, Ahmed A Bahrani, David K Powell, Larry B Goldstein, Gregory A Jicha

**Affiliations:** ^1^ University of Kentucky, LEXINGTON, KY, USA; ^2^ Sanders‐Brown Center on Aging, Lexington, KY, USA; ^3^ University of Kentucky College of Medicine, Lexington, KY, USA; ^4^ University of Kentucky, Lexington, KY, USA; ^5^ University of Kentucky Sanders‐Brown Center on Aging, Lexington, KY, USA

## Abstract

**Background:**

Cerebral amyloid angiopathy (CAA) is frequently associated with cognitive impairment and other types of dementia and can lead to lobar cerebral microbleeds and be associated with an increased burden of white matter hyperintensities (WMH). Traditional WMH quantification methods, such as simple volume subtraction, often fail to show the dynamic changes of WMH within subject, as they do not distinguish between WMH growth and regression. This study evaluates whether a novel longitudinal WMH growth and regression pipeline, validated within the MarkVCID consortium, can better differentiate CAA‐positive (CAA+) from CAA‐negative (CAA−) individuals.

**Method:**

Longitudinal (12‐month follow‐up) 3D FLAIR and T1‐weighted MRI scans (*n* = 78) from the University of Kentucky were analyzed using the WMH growth/regression pipeline to compute the WMH growth and regression volumes and location with subject over time. For comparison, conventional methods relying on absolute WMH volume subtraction were also applied. The participants scans were divided into two groups, CAA+(*n* = 29) and CAA− (*n* = 49) based on Bostin criteria. Statistical analyses were conducted to assess the ability of each approach to differentiate CAA+ from CAA− groups.

**Result:**

Only WMH growth was demonstrated a statistically significant difference between CAA+ and CAA− groups (*p* < 0.04), whereas conventional WMH volume subtraction failed to show a meaningful distinction (*p* = 0.067). This suggests that the dynamic nature of WMH growth, rather than static volumetric differences, is more sensitive to differentiate between the CAA+ and CAA‐ groups, unlike traditional approaches that combined both WMH changes growth and regression. Accordingly, WMH dynamic changes analysis, specifically WMH growth shows more sensitivity and provides a more precise marker of disease‐related changes.

**Conclusion:**

The WMH growth/regression pipeline serves as a novel, sensitive neuroimaging biomarker for distinguishing CAA+ from CAA− groups, compared to traditional volumetric methods. This approach enhances the accuracy of CAA diagnosis and deepens our understanding of cerebrovascular disease (CVD)‐related white matter pathology. Future research can expand the application of this method to other neurodegenerative disorders, including Alzheimer's disease, and explore its potential utility in clinical trials for CVD as a reliable imaging biomarker.